# Objective evaluation of fusional vergence after a vision therapy protocol in typical binocular vision

**DOI:** 10.1111/opo.13528

**Published:** 2025-05-24

**Authors:** Cristina Rovira‐Gay, Marc Argiles, Clara Mestre, Marta Masdemont‐Isern, Jaume Pujol

**Affiliations:** ^1^ Centre for Sensors, Instruments and Systems Development (CD6) Universitat Politècnica de Catalunya – BarcelonaTech (UPC) Barcelona Spain

**Keywords:** binocular vision, eye tracking, fusional vergence amplitudes, vision therapy

## Abstract

**Introduction:**

Vision therapy is an effective treatment option for binocular vision and accommodative anomalies. However, its effect on typical binocular vision is less documented. The aim of this study was to evaluate objectively the change in near fusional vergence amplitudes in adults with typical binocular vision after performing a vision therapy protocol.

**Methods:**

Thirty‐four adults were randomly classified into an experimental group (EG), who underwent a vision therapy protocol for 12 weeks, and a control group (CG), who received a placebo treatment for 12 weeks (Phase 1). In Phase 2, the CG performed the same therapy protocol as the EG. Fusional vergence amplitudes were measured objectively in a haploscopic set‐up using smooth and step fusional vergence tests. The break points of positive and negative fusional vergence (PFV and NFV, respectively) were determined using a custom algorithm.

**Results:**

After Phase 1, there were no significant differences between the groups for fusional vergences. After Phase 2, PFV and NFV increased significantly as measured with the smooth and step tests (*p* = 0.002 and *p* < 0.001 for NFV and *p* = 0.03 and *p* = 0.02 for PFV, respectively). These improvements were maintained after 1 month without any further intervention (all *p* > 0.05).

**Conclusion:**

Based on these results, clinicians should consider that the vergence system cannot be trained beyond 2 and 5 Δ for NFV and PFV, respectively, when aiming to enhance vergence skills with vision therapy in patients without binocular dysfunctions.

**Key points**

- After 12 sessions of vision therapy, both positive and negative fusional vergence amplitudes increased significantly.
- Asymptomatic participants with typical binocular vision exhibited a modest, yet clinically relevant, increase in fusional vergence amplitudes after completing a vision therapy protocol, highlighting the limits of vergence training in this population.
- Eye tracking systems can be used to measure vergence responses objectively, as well as monitor and assess the effects of vision therapy protocols.

## INTRODUCTION

Vision therapy is a sequence of neurosensory and neuromuscular exercises that are performed to develop, rehabilitate and improve visual skills and their processing.^1^ The literature extensively documents the occurrence of non‐strabismic binocular vision dysfunctions, although there is variability in the reported prevalence rates among studies, ranging from 13.5% to 32%.^2^, ^3^, ^4^, ^5^ Examining these cases, 2.29–15.81% were associated with accommodation disorders^2^, ^4^, ^5^ while 8–12.9% were attributed to vergence anomalies.^2^, ^6^ Convergence insufficiency (CI) is the most common non‐strabismic binocular dysfunction, with a prevalence that ranges from 2.25% to 17.6%.^7^, ^8^, ^9^, ^10^ CI is characterised by a larger exophoria at near than at distance, a receded near point of convergence and reduced positive fusional vergence (PFV) at near.^11^ One of the preferred treatment options for the management of CI is vision therapy, and its effectiveness has been well documented.^12^, ^13^ Vision therapy protocols for CI aim to achieve: (a) a near point of convergence of less than 6 cm, (b) PFV at near >15 prism dioptres (Δ) and satisfying Sheard's criterion, meaning that PFV is at least twice the magnitude of the heterophoria^14^ and (c) a symptom score < 16 on the CI symptom survey (CISS).^15^ However, previous studies have found that the CISS test might not be as sensitive to CI as expected because symptoms during near work can also be caused by other factors, for example, altered eye movements, accommodative disorders or other binocular vision dysfunctions, not exclusively CI.^16^, ^17^, ^18^ In addition, a study from the CITT‐ART Investigator Group found that CISS scores improved similarly in children with symptomatic CI who were randomised to the vergence/accommodative and placebo therapy groups. The authors suggested that the improvement in signs, rather than self‐reported symptoms, may be used as a measure of vision therapy success.^19^

Besides treating binocular vision disorders, vision therapy could also be used to improve the vergence skills of individuals with no binocular dysfunctions; however, its effect on typical binocular vision is less documented. These improved vergence skills might provide more comfortable and clear vision during tasks such as reading or using a computer, thereby reducing eye strain and fatigue.^20^ The consolidation of these skills not only might prevent potential future binocular vision problems but could also lead to better performance in academic, work and leisure activities. As the time spent viewing near distances has increased in recent years, a growing percentage of the population without binocular vision dysfunctions has begun to experience symptoms such as eye fatigue, eyestrain, headaches, ocular discomfort, dry eye, diplopia or blurred vision,^16^, ^21^ and the prevalence of computer vision syndrome has increased.^22^ Therefore, increasing fusional vergence ranges with vision therapy might overcome vision discomfort in individuals with typical binocular vision who have prolonged fixation at near distances.^23^ Additionally, increasing fusional vergence ranges and other oculomotor functions such as ocular motility could be useful to train vision abilities for activities such as e‐sports.^24^ Sports vision training is becoming more popular to enhance visual skills, improve eye–hand coordination, depth perception and reaction time.^25^ Visual parameters such as visual acuity, accommodative facility, near point of convergence or vergence facility have been found to be highly developed in athletes compared with non‐athletes,^26^ and these abilities can be trained with vision therapy. In addition, investigations using neuroimaging techniques such as functional magnetic resonance imaging (fMRI) have demonstrated changes in the activation of visual processing areas in response to visual therapy interventions.^27^, ^28^ However, the extent to which fusional vergence amplitudes can be improved in athletes with typical binocular and accommodative systems using conventional vision therapy protocols is not yet known.

The effects of vision therapy are typically assessed subjectively, because most clinical measures depend on the patients' responses or the experience of the examiners.^29^ Using conventional clinical methods, it is not possible to quantify the effects of vision therapy protocols objectively or generate objective records to monitor the change in oculomotor performance over time.^30^ Furthermore, symptom surveys may be affected directly by the placebo effect of therapy and subjective measures could be biased.^31^ In contrast, eye tracking systems can be used to measure vergence responses objectively and provide evidence of the change in eye movements with vision therapy. As this technology evolves and becomes more readily available in clinical settings, eye trackers could be used to monitor objectively and accurately the effects of vision therapy protocols, solving these current limitations.

Several studies have reported changes in the vergence responses of adults and children with CI measured with eye trackers after vision therapy.^29^, ^30^, ^32^ In a study of adolescents aged 12–17 years, there was a notable significant enhancement in peak velocity and greater precision in response amplitude when exposed to 4° symmetrical convergence step stimuli following an office‐based vergence/accommodative vision therapy (OBVAT) programme, compared with the initial baseline measurements. Additionally, the near point of convergence, PFV and symptomatology exhibited significant improvements after OBVAT.^30^ Furthermore, in a case report, the objective vergence outcome measures (peak velocity, time constant, total response time and steady‐state response variability) also improved after OBVAT in a 10‐year‐old participant.^32^ In addition, vergence eye movements have also been measured objectively following an OBVAT protocol in adults with concussion‐related CI, and improvement was reported at the end of the therapy.^29^

Talasan et al.^20^ performed an assessment procedure and vergence therapy protocol to stimulate disparity vergence while reducing the influence of proximal and accommodative vergence visual cues using a haploscopic system in a group of control participants. The evaluation quantified vergence latency, time to peak velocity, settling time, peak velocity and accuracy. When comparing the post‐vergence therapy outcomes with the baseline measures, the temporal and vergence accuracy values exhibited significant improvement.^20^

In the current study, participants with typical binocular vision performed a semi‐crossover OBVAT protocol and were divided into a control group (CG) and an experimental group (EG). The vision therapy protocol for the EG was designed to treat CI, as this is the therapy paradigm studied most frequently to train vergence. This programme has been proven to be effective in improving both convergence (or PFV) and divergence (or negative fusional vergence [NFV]) amplitudes in patients with CI.^14^, ^15^, ^30^, ^33^, ^34^ However, its effectiveness in improving the fusional vergence capabilities of individuals with typical binocular vision findings has not been well studied, and there is insufficient evidence regarding the impact of repetitive vergence therapy among individuals with typical binocular vision.^35^

The main goal of this study was to evaluate objectively the change in fusional vergence amplitudes at near in a group of adults with typical binocular and accommodative systems after performing an OBVAT protocol. In addition, a secondary goal was to assess objectively whether any effect of vision therapy on vergence movements persisted after 1 month without any intervention.

## METHODS

### Participants

A total of 32 adults between 19 and 29 years of age (mean ± standard deviation [SD]: 23.25 ± 3.14 years) with typical binocular vision participated in this semi‐crossover study. The required sample size was calculated using G*power software (psychologie.hhu.de/arbeitsgruppen/allgemeine‐psychologie‐und‐arbeitspsychologie/gpower)^36^ based on the PFV break point findings after a vision therapy protocol reported by Scheiman et al.^33^ The calculation indicated a sample size of 14 participants was required in each group to achieve a statistical power of 0.95. The inclusion and exclusion criteria are shown in Table 1.

**TABLE 1:** Inclusion and exclusion criteria.

Inclusion criteria
Age 18–30 years old Corrected visual acuity of 0.05 logMAR or better in both eyes at distance and near, using their habitual prescription Stereoacuity of at least 50 s of arc measured with the Random dot 2 steroacuity test (Bernell Corporation, bernell.com) at near No more than two of the following clinical signs^37^: exophoria of 10 Δ or greater at near, esophoria of 2 Δ or greater at near, near point of convergence break point >6 cm, reduced monocular amplitude of accommodation compared with the values predicted by Hofstetter's equation (18.5–0.25 × age), monocular and binocular accommodative facility with ±2.00 D lenses <7 cycles per minute (cpm), vergence facility with 3 Δ base‐in and 12 Δ base‐out prisms <9 cpm and fusional vergence ranges lower than Sheard's criterion, which states that the fusional reserve must be at least twice the magnitude of the heterophoria Convergence Insufficiency Symptom Survey^15^ score < 16 points to ensure that participants had no symptoms
Exclusion criteria
Previous history of vision therapy
Spherical refractive errors higher than +5.00 or −5.00 D; astigmatism >2.00 D or anisometropia >1.50 D
Presence of vertical heterophoria, ocular motility defects, nystagmus or strabismus
History of ocular pathology or ocular surgery
Use of active orthokeratology treatment
Use of any ocular or systemic medication known to affect accommodation, vergence or ocular motility

The study protocol was approved by the Ethics Committee of Hospital Mutua de Terrassa (Spain) and registered in the U.S. National Library of Medicine of Clinical Trials (registration code: NCT05208658). The study followed the tenets of the Declaration of Helsinki, and all subjects gave informed written consent after receiving a written and verbal explanation of the nature of the study.

### Study design and experimental procedure

**FIGURE 1:**
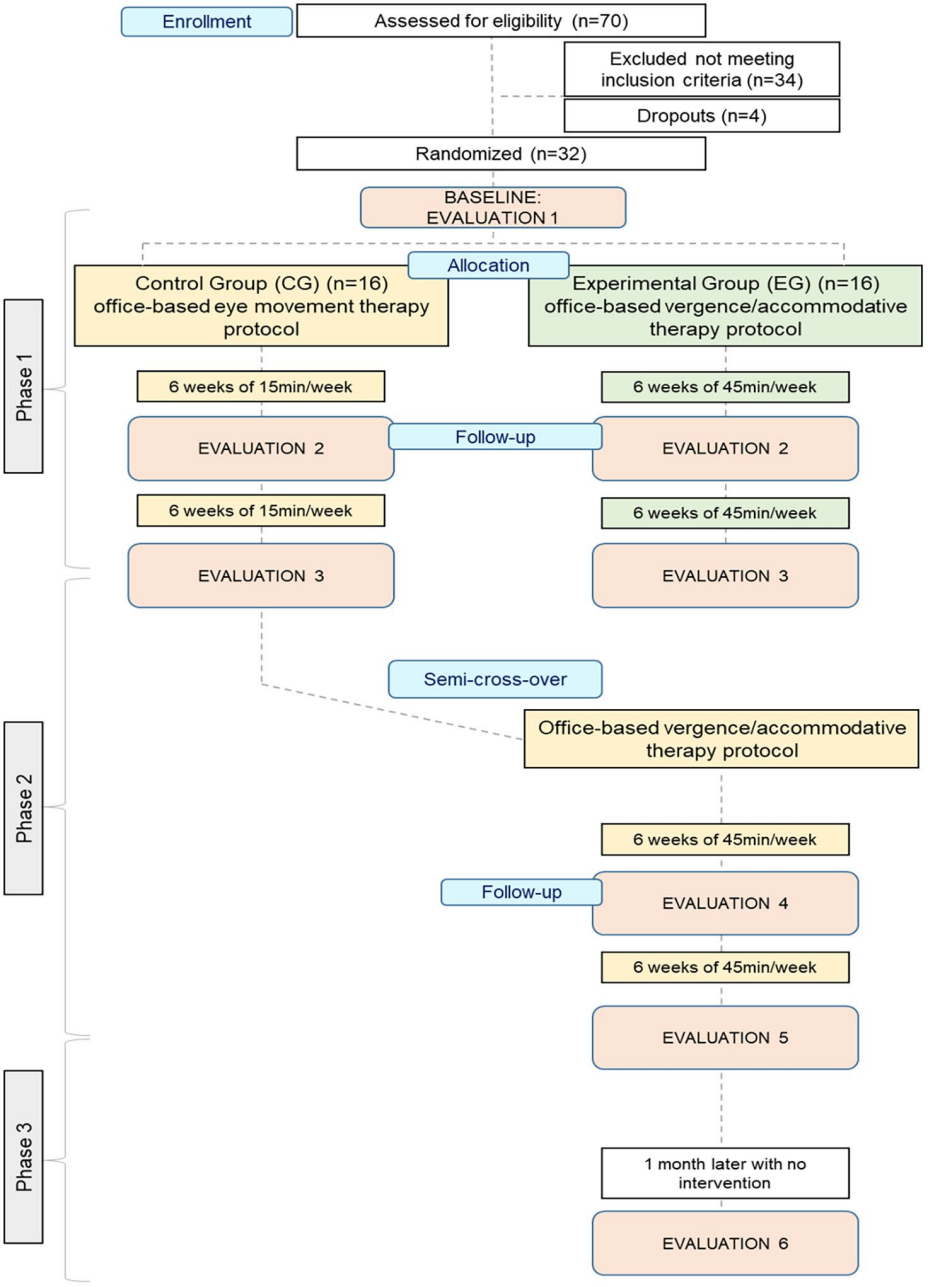
Flow diagram of the experimental design.

A randomised semi‐crossover, interventional and single‐blinded experimental design was carried out (Figure 1). The OBVAT protocol performed by participants in the EG consisted of 12 weeks (45 min/week) of conventional vergence therapy without home reinforcement. During these visits, participants completed three to five therapy procedures with constant supervision and guidance from an optometrist. The protocol followed the CITT Manual of Procedures for CI treatment (www.optometry.osu.edu/research/CITT/4363.cfm).^14^ A trained optometrist with experience in vision therapy followed this document, each procedure description, the amount of time used, expected performance and criteria for ending the procedure and advancing to a more difficult level.^12^ At the beginning of the protocol, instruments such as the Brock string or vectograms were used to perform easier exercises, while harder exercises such as the barrel card or the aperture rule were performed in the later sessions. This vision therapy protocol was designed to treat CI by training both PFV and NFV amplitudes to the same degree. Participants were not asked to perform reinforcement exercises at home as compliance could not be controlled.

The CG performed 12 weeks (15 min/week) of office‐based therapy without home reinforcement based on eye movement exercises in the frontal plane, with constant supervision and guidance from an optometrist. This customised protocol consisted of exercises that elicited smooth pursuit movements and visual perception exercises, such as visual discrimination tasks, performed at a constant viewing distance, which are not meant to stimulate the vergence and accommodation systems. For example, in the Hart Chart saccadic exercise, participants were asked to read letters from charts placed at 40 cm as quickly as possible. In the smooth pursuit exercises, participants followed the movement of a small object in different directions while maintaining the same distance or vergence demand.

After obtaining written consent, an eye examination was performed to determine whether the participants were eligible for the study. The battery of optometric tests included the same procedures as used in previous randomised clinical trials^31^, ^38^ and was performed using conventional standardised clinical techniques.^34^ This battery of tests included best‐corrected visual acuity at distance and near, both the cover‐uncover and the alternate cover tests at distance and near, monocular and binocular accommodative facility with ±2.00 D lenses, monocular amplitude of accommodation, near point of convergence, vergence facility at near with 3 Δ base‐in (BI) and 12 Δ base‐out (BO) prisms and PFV and NFV amplitudes at near. The monocular amplitude of accommodation and near point of convergence were measured three times and the results are presented as the mean of the three measures.

The measurement of fusional vergence amplitudes was of particular interest here because the break points of PFV and NFV were the main outcome variables used to determine the effect of vision therapy. They were evaluated subjectively and objectively using the step and smooth fusional vergence tests. The step subjective fusional vergence test was carried out using a prism bar with prism powers ranging from 1 to 40 Δ in the following steps: 1, 2, 4, 6, 8, 10, 12, 14, 16, 18, 20, 25, 30, 35 and 40 Δ. The prism bar was placed in front of the participant's right eye and the prism power was changed approximately every 2 s. Risley rotary prisms were used for the smooth subjective fusional vergence test. The prisms, mounted in a phoropter, ranged from 0 to 40 Δ and the prism power was changed at approximately 1 Δ/s. For the two subjective tests (step and smooth subjective tests), the break and recovery points were recorded as the prism power when participants reported diplopia and single vision while the vergence demand increased and decreased, respectively. The NFV amplitude was measured first with BI prisms to prevent vergence adaptation.^39^, ^40^ Then, the same procedure was repeated with BO prisms to measure the PFV amplitude.^41^ Three repetitions were performed for each fusional vergence test and the results were reported as the mean of the three measures.

Fusional vergence amplitudes were measured objectively in a haploscopic set‐up by recording eye movements with the eye tracker EyeLink 1000 Plus (SR Research, sr‐research.com) at 500 Hz. A column of 0.20 logMAR letters presented at each screen of the haploscope was used as a stimulus. The objective fusional vergence amplitudes were also measured with the smooth and step methods, as was the case for the conventional subjective tests and NFV amplitude, which were measured before PFV amplitude. In the smooth objective test, the stimuli moved synchronously in the two screens to increase the vergence demand at 1 Δ/s up to 45 Δ, to mimic the rotary Risley prisms. In the step objective test, the stimuli moved every 2 s in steps of 2 Δ from 2 to 20 Δ and steps of 5 Δ from 25 to 45 Δ. Three repetitions were performed consecutively for each vergence direction and objective test. In the objective tests, participants were not asked to report diplopia or single vision. A more detailed explanation of the haploscopic system, the calibration used, the experimental procedure and the validation of these objective tests to measure fusional vergences can be found in Rovira‐Gay et al.^42^

The study was divided into three phases, as shown in Figure 1. At the beginning of Phase 1 (P1), both EG and CG participants performed a baseline evaluation ( Evaluation 1 or E1), another evaluation in the middle of the protocol after six sessions of vision therapy (Evaluation 2 or E2), when EG and CG had already completed 4.5 and 1.5 h, respectively, and another at the end of the 12 weeks of vision therapy (Evaluation 3, E3), when the EG had undertaken 9 h of vision therapy and the CG 3 h. After E3, individuals in the EG finished their participation in the study, whereas participants in the CG started Phase 2 (P2), when they followed the same 12‐week OBVAT protocol as the EG had already completed. During Phase 2, the CG performed an evaluation at week 18 (Evaluation 4, or E4) and a final evaluation at week 24 (Evaluation 5, or E5). In Phase 3 (P3), the CG group undertook another evaluation (Evaluation 6, E6), 1 month after finishing the vision therapy protocol to assess the persistence of the effects over time.

The same battery of optometric tests used to determine participants' eligibility as well as subjective and objective fusional vergence amplitude measurements were carried out in each evaluation (E1–E6) for all participants. Results from the objective assessment of fusional vergence amplitudes are included in the main Results section, as this was the primary objective of the study. Fusional vergence amplitudes measured subjectively using conventional procedures are also reported for comparison. The results of all other optometric tests at each evaluation are reported in Table S1 (Phase 1, with results of E1, E2 and E3) and S2 (Phase 2 and Phase 3 with results of E4, E5 and E6).

### Data analysis

The objective fusional vergence break and recovery points in each repetition were determined offline using a custom algorithm written in MATLAB (mathworks.com) for analysis of the eye tracking recordings.^42^ For this study, only the break points were included in the analysis. First, periods of 200 ms before and after each blink, as detected by the EyeLink software, were removed from the signal and filled by linear interpolation to avoid artefacts associated with the onset and offset of blinks. Fusional vergence break points were determined using an iterative least‐squares fitting procedure. For the smooth objective test, a straight line was fitted to the measured vergence position over time iteratively, adding 0.10 s of data in each iteration. For the step objective test, a second‐degree polynomial function was fitted instead of a line, as it described better the change in vergence demand over time. Then, the break point was determined as the vergence demand at the time of the last fit before the coefficient of determination of the fit started to decrease.

A break point of 45 Δ, the maximum vergence demand stimulated, was assigned to participants who did not exhibit loss of motor fusion during the objective tests, while a break point of 40 Δ, the maximum prism power of the Risley prisms and the prism bar, was assigned to those who did not report diplopia during the subjective tests.

IBM SPSS software 27.0 (ibm.com) was used for statistical analysis. A significance level (*p*‐value) < 0.05 was considered statistically significant. All variables were first examined for normality using the Shapiro–Wilk test. Parametric tests were used with normally distributed variables, while nonparametric tests were used when normality could not be assumed.

For Phase 1, a mixed ANOVA with one within‐subjects factor (time; evaluations 1, 2 and 3) and one between‐subjects factor (group; experimental or control) was used to determine the differences in fusional vergence break points measured at evaluations 1–3 between the CG and EG (interaction term between time and group). Post hoc tests with Bonferroni correction were carried out for the significant interactions. For Phase 2, a repeated measures ANOVA with Bonferroni post hoc test was used to determine whether there were significant changes in fusional vergence break points measured at evaluations 3, 4 and 5. For Phase 3, paired *t*‐tests or Wilcoxon signed‐rank tests were used to determine the differences in fusional vergence break points measured at evaluations 5 and 6, after 1 month with no intervention.

## RESULTS

Participants were randomly assigned to the experimental and control groups (16 participants in each group, 14 and 9 females in the EG and CG groups, respectively). The mean ± SD age was 21.31 ± 1.74 years for the EG and 25.18 ± 3.05 years for the CG.

The baseline results of the binocular vision tests performed before starting the vision therapy protocol (P1–E1) for the EG participants and the CG participants are presented in Table 2 (all *p* > 0.05).

**TABLE 2:** Mean (±SD) of baseline data.

Optometric test	Experimental group	Control group
Heterophoria at 40 cm	1 Δ exophoria ± 5 Δ	0 Δ ± 1 Δ
Near point of convergence: break point	6 ± 1 cm	9 ± 5 cm
Near point of convergence: recovery point	7 ± 1 cm	11 ± 5 cm
Binocular accommodative facility	10 ± 3 cpm	11 ± 2 cpm
Vergence facility	18 ± 5 cpm	17 ± 5 cpm

Abbreviations: cpm, cycles per minute; Δ, prism diopters.

The results of all optometric tests performed at each evaluation are presented in Table S1 (Phase 1, with results of E1, E2 and E3) and S2 (Phase 2 and Phase 3 with results of E4, E5 and E6).

Before starting the vision therapy protocols, the two groups also exhibited similar fusional vergence amplitudes, as there were no significant differences between the groups for the NFV break point of the smooth subjective test (*t*(15) = −1.23, *p* = 0.24), step subjective test (*z* = − 0.35, *p* = 0.72), smooth objective test (*t*(15) = −0.57, *p* = 0.58) and step objective test (*t*(15) = −0.57, *p* = 0.58). Similarly, there were no significant differences between the PFV break point for the CG and the EG groups measured with the smooth and step subjective tests (*t*(15) = − 1.96, *p* = 0.07 and *z* = −0.82, *p* = 0.41, respectively) and the smooth and step objective tests (*z* = −0.45, *p* = 0.65 and *z* = −0.47, *p* = 0.64, respectively).

**FIGURE 2:**
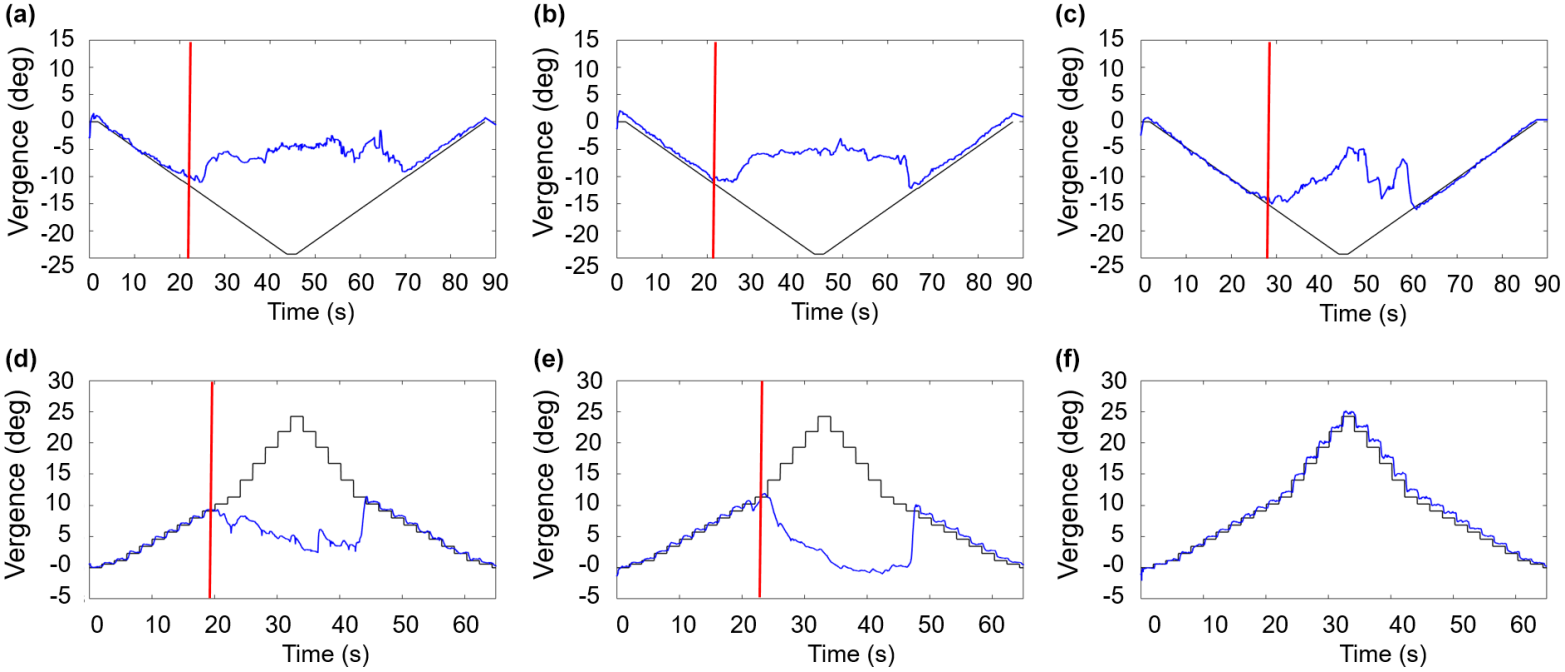
Examples of representative eye tracking recordings. (a–c) Recordings during negative fusional vergence (NFV) measurements with the smooth objective test for one representative participant in evaluations 1 (Phase 1), 3 (Phase 2) and 5 (Phase 3), respectively. (d–f) Recordings during positive fusional vergence (PFV) measurements with the step objective test for a different participant in the same evaluations (evaluations 1 (Phase 1), 3 (Phase 2) and 5 (Phase 3), respectively). (f) An example of a recording where the participant did not exhibit loss of motor fusion and was assigned a break point of 45 Δ. The red lines represent the break points determined objectively, vergence demand over time is represented in black and measured vergence position in blue.

**FIGURE 3:**
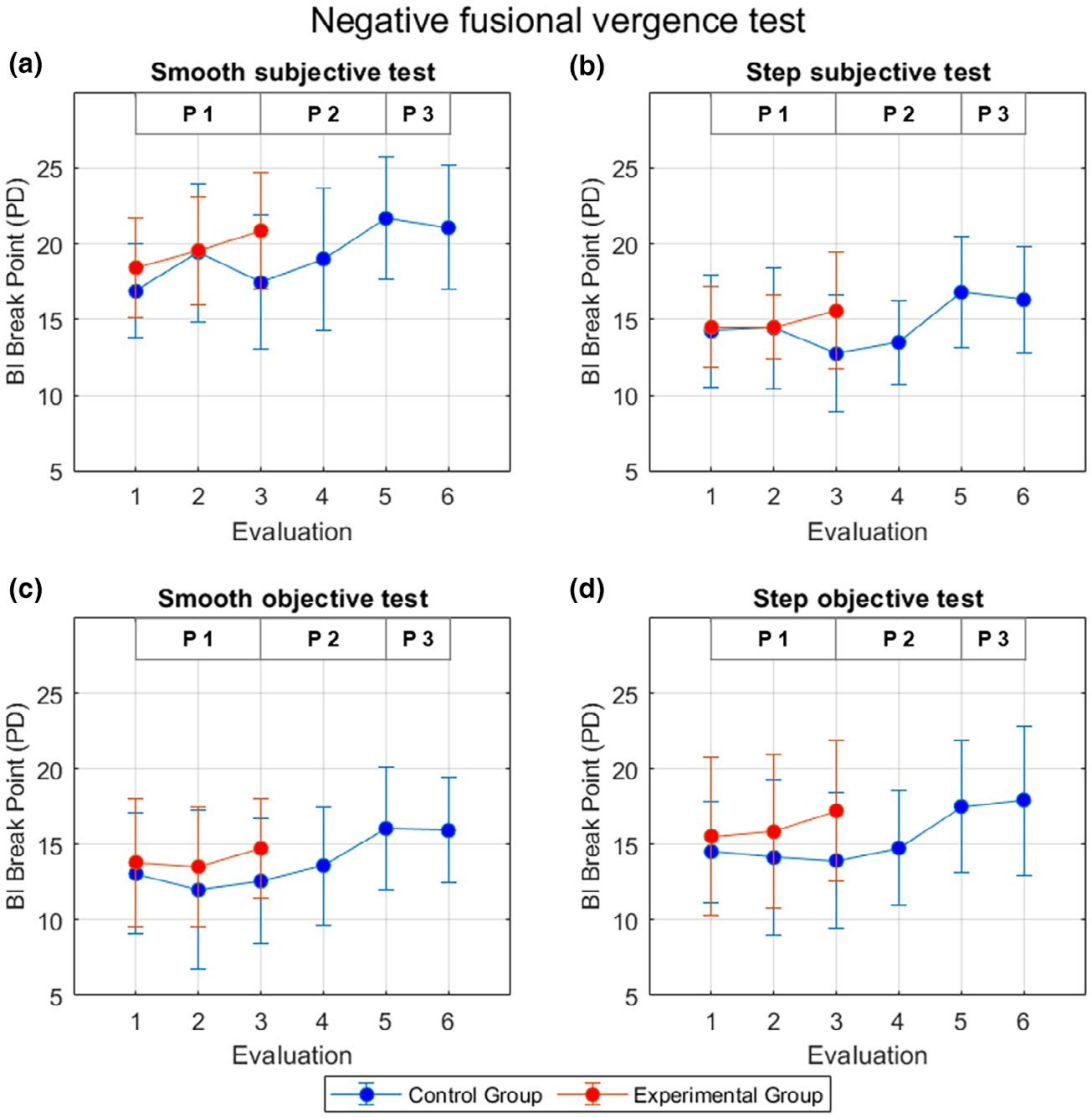
Mean ± SD negative fusional vergence (NFV) amplitudes across participants in the control (blue) and experimental groups (red) measured at each evaluation with the smooth and step subjective tests (a, b) and the smooth and step objective tests (c, d). The three Phases (P1, P2 and P3) are identified in each panel. PD, prism dioptres.

**FIGURE 4:**
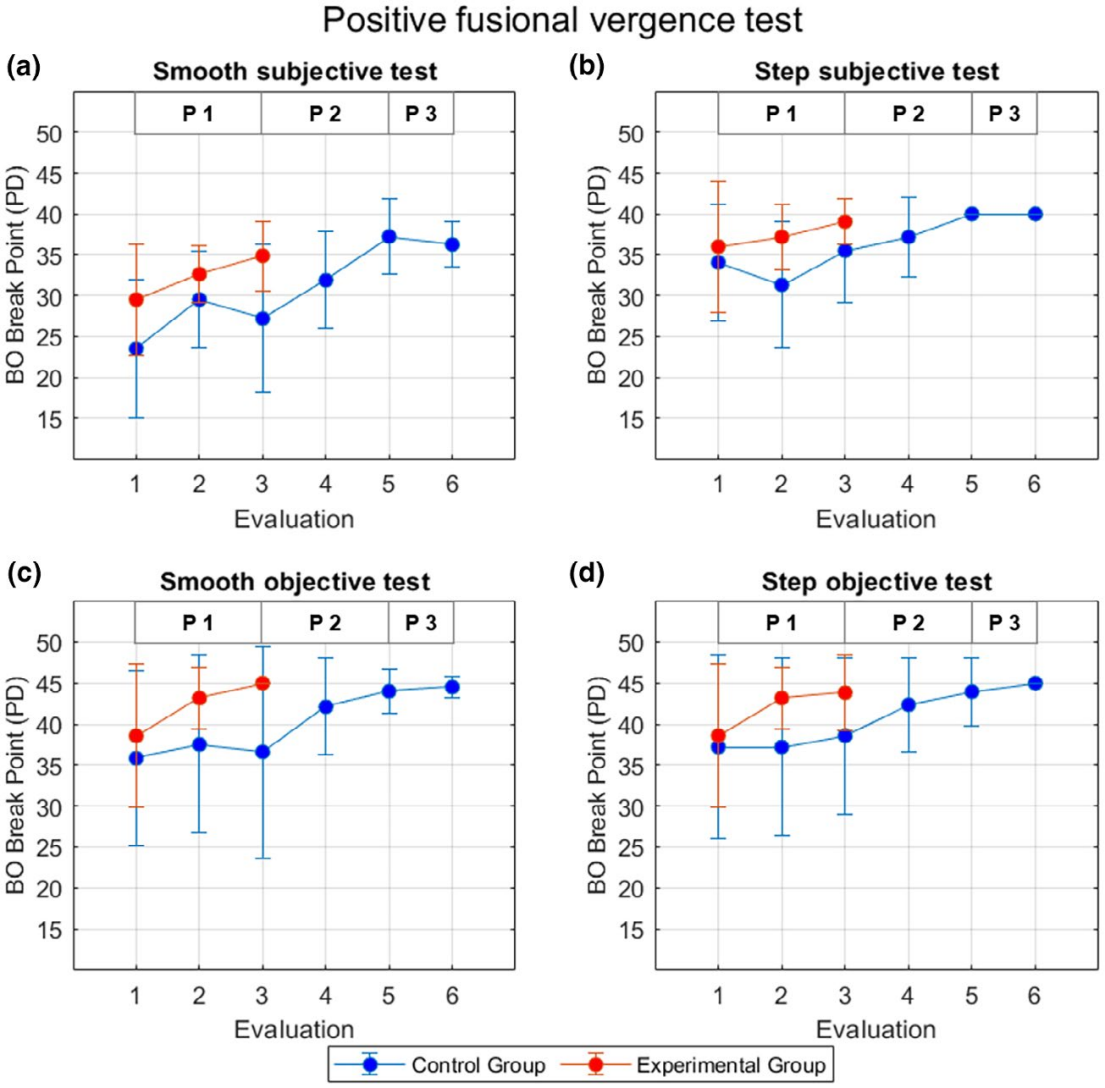
Mean ± SD positive fusional vergence (PFV) amplitudes across participants in the control (blue) and experimental groups (red) measured at each evaluation with the smooth and step subjective tests (a, b) and the smooth and step objective tests (c, d). The three Phases (P1, P2 and P3) are identified in each panel. PD, prism dioptres.

Figure 2 shows examples of eye tracking recordings of two representative participants obtained at E1, E3 and E5 during the performance of the smooth and step objective tests. Mean (and SD) NFV amplitudes across participants measured at each evaluation are presented in Figure 3, while the mean (and SD) PFV amplitudes are shown in Figure 4.

### Phase 1

The mean (±SD) fusional vergence amplitudes of both groups, measured with the four tests in the first three evaluations (E1–E3) are shown in Table 3.

**TABLE 3:** Mean (±SD) NFV and PFV amplitudes measured with the smooth and step subjective and objective tests at evaluation 1 (E1), evaluation 2 (E2) and evaluation 3 (E3) of Phase 1 for both the control and experimental groups.

Vergence measure (Δ)		Control group			Experimental group			*p*‐Value
		E1	E2	E3	E1	E2	E3	
NFV break	Smooth subjective	16.87 ± 3.09	19.43 ± 4.56	17.43 ± 4.41	18.43 ± 3.28	19.56 ± 3.55	20.87 ± 3.86	0.007*
	Step subjective	14.25 ± 3.71	14.43 ± 3.98	12.75 ± 3.85	14.50 ± 2.68	14.50 ± 2.12	15.56 ± 3.84	0.10
	Smooth objective	13.06 ± 4.01	11.98 ± 5.27	12.58 ± 4.17	13.80 ± 4.22	13.53 ± 3.93	14.74 ± 3.27	0.34
	Step objective	14.52 ± 3.31	14.14 ± 5.13	13.93 ± 4.50	15.54 ± 5.19	15.84 ± 5.09	17.22 ± 4.63	0.12
PFV break	Smooth subjective	23.50 ± 8.50	29.50 ± 5.93	27.18 ± 9.08	29.50 ± 6.74	32.62 ± 3.46	34.87 ± 4.25	0.15
	Step subjective	34.06 ± 7.12	31.31 ± 7.68	35.50 ± 6.37	36.00 ± 8.04	37.18 ± 4.06	39.06 ± 2.71	0.34
	Smooth objective	35.84 ± 10.70	37.52 ± 10.86	36.60 ± 12.91	38.57 ± 8.80	43.17 ± 3.74	45.00 ± 0.00	0.07
	Step objective	37.20 ± 11.21	37.20 ± 10.89	38.58 ± 9.54	38.79 ± 8.05	43.22 ± 3.77	43.83 ± 4.66	0.22

Abbreviations: E, evaluation; NFV, negative fusional vergence; PFV, positive fusional vergence; Δ, prism dioptres.

* A statistically significant interaction between the evaluations and groups (*p* < 0.05).

Considering the results obtained in the first three evaluations (9 h of therapy for EG and 3 h of therapy for CG), there was no significant effect of vision therapy on NFV amplitudes as shown by the absence of significant interactions between the evaluation and group factors, except for the NFV amplitudes measured with the smooth subjective test (*F*(2, 60) = 5.42, *p* = 0.007, ŋ^2^ = 0.15). In this case, the post hoc Bonferroni analyses for pairwise comparisons revealed significant differences between groups at E3 (*p* = 0.03), but no statistically significant differences at E1 (*p* = 0.18) and E2 (*p* = 0.93). Considering the simple main effect of time (evaluations), paired *t*‐tests with Bonferroni correction (significance level set at *p* < 0.016) were performed to assess differences between each evaluation for each group separately. The control group exhibited significant differences between E1 and E2 (*p* < 0.001), but not between E2 and E3 (*p* = 0.07) or E1 and E3 (*p* = 0.55). In contrast, the experimental group did show significant differences in NFV break points measured with the smooth subjective test in E1 and E3 (*p* = 0.004), but not between E1 and E2 (*p* = 0.05) or E2 and E3 (*p* = 0.11). The change in NFV amplitudes as a function of the group measured with the other tests did not reach statistical significance [*F*(2, 60) = 2.43, *p* = 0.10, ŋ^2^ = 0.08; *F*(2, 60) = 1.10, *p* = 0.34, ŋ^2^ = 0.04; and *F*(2, 60) = 2.20, *p* = 0.12, ŋ^2^ = 0.07, for the step subjective test, smooth objective test and step objective test, respectively]. Similarly, although on average PFV amplitudes increased more for the EG than the CG throughout Phase 1 (Figure 4), the interaction between the two factors did not reach statistical significance [*F*(1.63, 49.16) = 2.02, *p* = 0.15, ŋ^2^ = 0.06; *F*(1.43, 42.90) = 1.04, *p* = 0.34, ŋ^2^ = 0.03; *F*(1.39, 41.95) = 3.15, *p* = 0.07, ŋ^2^ = 0.10 and *F*(1.59, 47.69) = 1.58, *p* = 0.22, ŋ^2^ = 0.05, for the smooth subjective, step subjective, smooth objective and step objective tests, respectively].

### Phase 2

After week 12, the CG started the semi‐crossover part of the study, when participants performed the same OBVAT protocol as the EG in Phase 1 following the same procedures (9 h of vision therapy). The mean and SD fusional vergence amplitudes of the CG measured with the four tests at E3 and E5 are shown in Table 4 and Figures 3 and 4.

**TABLE 4:** Mean (±SD) NFV and PFV amplitudes measured with the smooth and step subjective and objective tests at E3 to E5 (Phase 2) and E6 (Phase 3).

Vergence measure (Δ)		Evaluation 3	Evaluation 4	Evaluation 5	*p*‐Value	Evaluation 6	*p*‐Value
NFV break	Smooth subjective	17.73 ± 4.41	19.00 ± 4.70	21.68 ± 4.04	<0.001*	21.06 ± 4.09	0.35
	Step subjective	12.75 ± 3.85	13.50 ± 2.78	16.81 ± 3.63	<0.001*	16.31 ± 3.49	0.77
	Smooth objective	12.58 ± 4.17	13.60 ± 3.90	16.05 ± 4.02	0.002*	15.93 ± 3.47	0.77
	Step objective	13.93 ± 4.50	14.76 ± 3.83	17.51 ± 4.35	<0.001*	17.93 ± 4.94	0.34
PFV break	Smooth subjective	27.18 ± 9.09	31.87 ± 5.90	37.25 ± 4.55	<0.001*	36.25 ± 2.81	0.26
	Step subjective	35.50 ± 6.37	37.18 ± 4.81	40.00 ± 0.00	0.01*	40.00 ± 0.00	–
	Smooth objective	36.60 ± 12.91	42.10 ± 5.90	44.00 ± 2.75	0.03*	44.51 ± 1.27	0.45
	Step objective	38.58 ± 9.54	42.35 ± 5.70	43.95 ± 4.16	0.02*	45.00 ± 0.00	–

Abbreviations: E, evaluation; NFV, negative fusional vergence; PFV, positive fusional vergence; Δ, prism dioptres.

* A statistically significant difference between evaluations (*p* < 0.05). For the two step tests, no statistical analysis was performed on participants who achieved the maximum vergence demand stimulated (see details in text).

In the semi‐crossover part of the study, both NFV and PFV amplitudes measured with all four tests showed statistically significant differences, indicating a fusional vergence improvement after performing the OBVAT therapy protocol. The repeated measures ANOVA showed statistically significant differences between E3 and E5 (Phase 2) for NFV amplitudes measured with the smooth subjective test (*F*(2, 30) = 10.90, *p* < 0.001, ŋ^2^ = 0.42). The post hoc Bonferroni analysis demonstrated that the significant differences were found between E3 and E5 (*p* = 0.006) and E4 and E5 (*p* = 0.009), but not between E3 and E4 (*p* = 0.22). Similarly, statistically significant differences in NFV as measured with the step subjective test, smooth and step objective tests were found between E3 and E5 (*F*(2, 30) = 12.43, *p* < 0.001, ŋ^2^ = 0.45; *F*(2, 30) = 7.41, *p* = 0.002, ŋ^2^ = 0.33; and *F*(2, 30) = 9.69, *p* < 0.001, ŋ^2^ = 0.39, respectively). Post hoc analyses also showed that these differences were between E3 and E5 (*p* < 0.001, *p* = 0.03 and *p* = 0.009, respectively) and E4 and E5 (*p* < 0.001, *p* = 0.03 and *p* = 0.023, respectively), whereas no statistically significant differences were found between E3 and E4 (*p* > 0.99, *p* = 0.55 and *p* = 0.57, respectively).

For the measurement of PFV amplitudes, the results of all tests also showed statistically significant differences between E3 and E5 (Phase 2). For the smooth subjective test, the statistically significant differences found in Phase 2 (*F*(2, 30) = 15.26, *p* < 0.001, ŋ^2^ = 0.50) were between E3 and E5 (*p* < 0.001) and E4 and E5 (*p* = 0.008), as shown by the post hoc analysis. However, for the step subjective test, the statistically significant difference found with the repeated measures ANOVA (*F*(2, 30) = 5.02, *p* = 0.01, ŋ^2^ = 0.25) was between E3 and E5 (*p* = 0.04). In contrast, for the smooth and step objective tests, although a statistically significant change in PFV amplitudes was found in Phase 2 [*F*(1.16, 17.41) = 5.20, *p* = 0.03, ŋ^2^ = 0.26; and *F*(1.47, 22.08) = 5.19, *p* = 0.02, ŋ^2^ = 0.26, respectively], no statistically significant differences were found between pairs of evaluations in the post hoc analyses.

### Phase 3

One month after completing the vision therapy protocol, NFV and PFV amplitudes measured with any of the four tests were not statistically significantly different as shown by paired *t*‐tests or Wilcoxon tests when comparing the results obtained at E5 and E6 (Phase 3). This means that the effects of vision therapy were maintained over time. Specifically, no statistically significant differences in NFV amplitudes, as measured with the smooth and step subjective tests, were found (U = −0.94, *p* = 0.35; and *t*(15) = −0.38, *p* = 0.71, respectively), either between the amplitudes measured with the smooth or step objective tests (*t*(15) = 0.30, *p* = 0.77 and *t*(15) = −0.99, *p* = 0.34, respectively). For PFV amplitudes measured with the smooth subjective and objective tests, the differences between E5 and E6 were not statistically significant (*t*(15) = 1.17, *p* = 0.26 and *t*(15) = −0.78, *p* = 0.45, respectively). Most participants were assigned the maximum PFV value for the step subjective and both objective tests in E5 and E6 as they did not report diplopia or exhibit loss of motor fusion, as represented in Figure 2f. For this reason, the differences between these two evaluations could not be analysed statistically.

## DISCUSSION

In this randomised semi‐crossover, interventional experimental study, fusional vergence amplitudes were measured objectively and subjectively in participants with typical binocular vision after performing 9 h of an OBVAT protocol.

Participants of this study did not have any significant binocular vision‐related alteration, although they could have up to two clinical signs as described in the inclusion criteria (Table 1). These inclusion criteria were the same used in previous clinical trials^37^ and followed Morgan's expected findings to identify normal binocular and accommodative participants.^11^ Moreover, they all had a normal CISS score and they did not manifest vision‐related symptomatology. Although there is considerable controversy regarding the effectiveness of the CISS, participants in this study did not manifest fatigue after reading nor did they exhibit any symptomatology.

After Phase 1 (9 h of vergence treatment), on average, fusional vergence amplitudes increased in the EG. In general, the change throughout the first three evaluations (E1–E3) was not significantly different between the EG and CGs. Incidentally, the NFV amplitude measured with the smooth subjective test was the only outcome variable for which the improvement shown by the EG was significantly different from that exhibited by the CG after the first 12 weeks of the experimental protocol (*p* = 0.007). Interestingly, post hoc analyses showed that the difference between the groups was statistically significant only at E3 and not in the baseline evaluation (E1) nor the first follow‐up after beginning the protocol (E2). Besides the statistical findings, the results of this study should be interpreted based on clinical relevance. Although the effect of vision therapy in Phase 1 did not reach statistical significance for most of the variables, probably due to the high between‐subjects variability, on average, there was an increase of >2 Δ (or even >5 Δ) for PFV amplitudes measured with most of the variables (Table 3). Usually, a difference of 2 Δ is considered clinically relevant,^43^ so the effect of the OBVAT protocol observed in the current study could be considered clinically significant.

Previous studies of children diagnosed with CI reported that significant improvements in convergence measures (PFV and near point of convergence) can be achieved at 4 weeks (9 h) of OBVAT.^44^ In addition, Scheiman et al.^45^ in a study of children with CI, found mean NFV and PFV amplitudes of 19.6 and 38.3 Δ, respectively, when measured with the step test after 12 weeks (27 h) of OBVAT with home reinforcement. These results are comparable with the values obtained in the current investigation at Evaluation 3 (Phase 1), with mean NFV amplitudes ranging between 14.74 and 20.87 Δ and mean PFV amplitudes between 34.87 and 45.00 Δ, depending on the measurement test being used. Despite the similar results after 9 h of OBVAT, the fact that baseline fusional vergence amplitudes were already relatively high in the current investigation limited the statistical significance of clinically relevant improvements of around 2 and 5 Δ in NFV and PFV, respectively. Similar changes in fusional vergence amplitudes after 12 h of OBVAT with home reinforcement were described by Sangoi et al. in a sample of adult participants with typical binocular vision.^46^ These results suggest the existence of a limit of fusional vergence amplitudes, after which the visual system cannot increase its capacity further. Moreover, other aspects that should be taken into account are the fact that the participants were adults, who might exhibit limited neural plasticity compared with children,^47^ and that fewer hours of vision therapy were performed as they did not do home reinforcement therapy. While most studies of vision therapy are conducted in children,^31^, ^48^ some studies in adults with CI showed similar improvement in fusional vergence amplitudes.^46^

In Phase 2 (E3–E5), participants initially allocated to the CG who undertook 9 h of the experimental OBVAT protocol showed an improvement in fusional vergence amplitudes for both NFV and PFV measured with all tested methods. In this phase, the change was statistically significant. Moreover, an additional mixed ANOVA was performed to compare the rate of change in fusional vergence amplitudes of the EG during Phase 1 (E1–E3) and the CG during Phase 2 (E3–E5) (different evaluation numbers but same time points along the OBVAT protocol) and no significant differences were found (all *p* > 0.05). This suggests that the effect of the vision therapy on fusional vergence amplitudes was similar in the two groups.

A certain asymmetry was found between the improvement in NFV and PFV amplitudes. For NFV amplitudes, statistically significant differences were found in Phase 2, between E3 and E5 and E4 and E5 (after 6 or 9 h of therapy, respectively) for all of the methods evaluated (step and smooth objective and subjective tests). Similar results were noted for PFV amplitudes measured with the smooth subjective test. With the step subjective test, significant differences were only observed after 9 h of therapy (E3 and E5) and no significant change in PFV amplitudes was recorded between any evaluation pair. Erkelens and Bobier suggested that divergence and convergence mechanisms have strong directional asymmetries in disparity‐driven vergence responses, as they are controlled by different neural mechanisms and innervated by varying different neural pathways.^49^ This highlights that convergence and divergence have different adaptation capacities, which could lead to different improvements after treatment. In particular, it tends to be more difficult to train divergence than convergence eye movements and, as a result, the effects of vision therapy tend to be more modest for NFV.^48^, ^50^ Representative examples of this effect are illustrated in Figure 2. In the current study, another source of asymmetry in the effect of vision therapy on fusional vergence amplitudes, yet in the opposite direction, was a ceiling effect in the measurement of PFV amplitudes. As shown in Figure 4 and Tables 3 and 4, at the end of the OBVAT protocols (either in Evaluation 3 or Evaluation 5), most, if not all, of the participants did not report diplopia or exhibit loss of motor fusion during the PFV amplitude measures and were assigned a break point value of 40 Δ for the subjective tests or 45 Δ for the objective measurements. Consequently, whereas these measurements could capture completely the effect of the OBVAT protocol on NFV amplitudes, it is feasible that the observed change in PFV could have been greater with an extended measurement range, as participants reached the upper limit of both tests.^51^ However, the functional advantage of having PFV amplitudes of 45 Δ or greater is debatable.

After a month (Phase 3, E5–E6), there were no statistically significant differences in fusional vergence amplitudes measured with any of the tests, which indicated that the effects of vision therapy were stable over at least this duration. This finding is comparable with the results found by the CITT study group, who observed that the improvements in symptoms and signs persisted after a month and at least for 1 year after finishing a vision therapy treatment.^52^ A limitation of the present study is that the experimental group was not evaluated 1 month after completing the vision therapy protocol. However, it is unlikely that significant statistical differences would have been observed, given that no significant differences were detected among Phases 1, 2 and 3 in the first place, suggesting stability in the measured outcomes over time, even during the vision therapy protocol. Additionally, data regarding the symptomatology of participants were not collected throughout the vision therapy protocol. Motivation and management of participants' expectations are two factors which are subjective and could have impacted the results of the study.^53^ In an attempt to control for these factors, the same trained optometrist guided the vision therapy protocols, attempting to encourage the same level of motivation for all participants. Although motivation could differ between individuals, the cross‐over study design allowed the description of a qualitatively similar effect of vision therapy in the two groups of participants. The protocol did not include home reinforcement^54^ as it was not possible to monitor motivation and compliance at home. Although the CG participants were likely aware that their first 12 sessions (Phase 1) were shorter than those in Phase 2, the duration of all eye movement exercises during vision therapy was recorded. Throughout these weeks, participants focused on improving their execution time, which fostered a sense of progress and enhanced their motivation, ensuring that all potential biases remained masked. In addition, the study was only single blinded, and despite this limitation, efforts were made to mitigate potential biases through the study design and data analysis procedures. It should be noted that only the subjective fusional vergence measures could have been biased by the unmasked examiner. The objective fusional vergence amplitudes were estimated from the offline analysis of eye tracking recordings.

The effects of the vision therapy protocol assessed with objective tests were comparable with those assessed with the subjective tests. Although the measures obtained with the four different tests did not always agree, as shown previously by other studies,^42^, ^51^, ^53^, ^55^, ^56^ a similar rate of change in fusional vergence amplitude along the vision therapy protocol was captured with the four tested methods. Therefore, eye tracking systems could be a useful tool in vision therapy to provide clinicians with objective data regarding their patients' visual abilities and can be used to develop and implement effective treatment plans, as well as monitor progress in an objective manner. The next step of this work is to study the dynamics of fusional vergence, for example, gain, latency and velocity, before and after a vision therapy protocol to offer new insights into the vergence mechanisms that are changed with the intervention in individuals with typical binocular vision.

To conclude, this study provides new insight into the possibility of training fusional vergence amplitudes in participants with typical binocular vision by providing objective evidence of the effects of vision therapy. After 9 h of an OBVAT protocol, modest clinically relevant improvements in fusional vergence amplitudes were found. The results of the present study suggest that, in asymptomatic individuals with typical binocular and accommodative systems, additional improvements may be restricted by the inherent neural adaptability of the vergence system, which may prevent excessive modifications beyond its natural capacity. Clinicians may consider these results in future interventions before training vergence skills in asymptomatic patients with good visual ability, as may be when training vergence ranges in sports vision, or in cases of ocular symptomatology due to digital eye strain without other ocular dysfunctions.

## AUTHOR CONTRIBUTIONS

**Cristina Rovira‐Gay:** Conceptualization (lead); data curation (lead); formal analysis (equal); investigation (equal); methodology (equal); software (equal); writing – original draft (lead); writing – review and editing (equal). **Marc Argiles:** Conceptualization (equal); data curation (equal); investigation (equal); methodology (equal); supervision (equal); writing – review and editing (equal). **Clara Mestre:** Conceptualization (equal); investigation (equal); software (equal); supervision (equal); writing – review and editing (equal). **Marta Masdemont‐Isern:** Data curation (supporting); investigation (supporting); methodology (supporting); writing – review and editing (supporting). **Jaume Pujol:** Funding acquisition (lead); project administration (lead); supervision (supporting); writing – review and editing (equal).

## CONFLICT OF INTEREST STATEMENT

The authors have declared that no competing interests exist.

## FUNDING INFORMATION

This publication is part of the project PID2020‐112527RB‐I00, PID2023‐146101OB‐I00 and TED2021‐130409B‐C54, funded by MCIN/AEI/10.13039/501100011033 and the European Union ‘NextGenerationEU’/PRTR.

## Supporting information


Data S1.

## References

[1] Williams GJ , Cotter SA , Frantz KA , Hoffman LG , Steele MGT , Miller SC , et al. The primary eye care profession: care of the patient with learning related vision problems. Am Optom Assoc. 2000;1–38.

[2] García‐Muñoz Á , Carbonell‐Bonete S , Cantó‐Cerdán M , Cacho‐Martínez P . Accommodative and binocular dysfunctions: prevalence in a randomised sample of university students. Clin Exp Optom. 2016;99:313–321. 27027297 10.1111/cxo.12376

[3] Franco S , Moreira A , Fernandes A , Baptista A . Accommodative and binocular vision dysfunctions in a Portuguese clinical population. J Optom. 2022;15:271–277. 34852966 10.1016/j.optom.2021.10.002 PMC9537267

[4] Shrestha P , Kaiti R . Non‐strabismic binocular vision dysfunction among the medical students of a teaching hospital: a descriptive cross‐sectional study. J Nepal Med Assoc. 2022;60:693–696. 10.31729/jnma.7615 PMC9446491 36705215

[5] Jang JU , Park IJ . Prevalence of general binocular dysfunctions among rural schoolchildren in South Korea. Taiwan J Ophthalmol. 2015;5:177–181. 29018694 10.1016/j.tjo.2015.07.005 PMC5602136

[6] Lara F , Cacho P , García Á , Megías R . General binocular disorders: prevalence in a clinic population. Ophthalmic Physiol Opt. 2001;21:70–74. 11220042 10.1046/j.1475-1313.2001.00540.x

[7] Hussaindeen JR , Rakshit A , Singh NK , George R , Swaminathan M , Kapur S , et al. Prevalence of non‐strabismic anomalies of binocular vision in Tamil Nadu: report 2 of BAND study. Clin Exp Optom. 2017;100:642–648. 27859646 10.1111/cxo.12496

[8] Davis AL , Harvey EM , Twelker JD , Miller JM , Leonard‐Green T , Campus I . Convergence insufficiency, accommodative insufficiency, visual symptoms and astigmatism in Tohono O'odham students. J Ophthalmol. 2016;2016. 10.1155/2016/6963976 PMC4971328 27525112

[9] Wajuihian SO , Hansraj R . Vergence anomalies in a sample of high school students in South Africa. Aust J Optom. 2016;9:246–257. 10.1016/j.optom.2015.10.006 PMC5030317 26750804

[10] Porcar E , Martinez‐Palomera A . Prevalence of general binocular dysfunctions in a population of university students. Optom Vis Sci. 1997;74:111–113. 9097328 10.1097/00006324-199702000-00023

[11] Scheiman MM , Wick B . Clinical Management of Binocular Vision. Heterophoric, accommodative and eye movement disorders. Philadelphia, Pennsylvania: Lippincott Williams & Wilkins; 2014. p. 247–249.

[12] Scheiman MM , Gwiazda J , Li T . Non‐surgical interventions for convergence insufficiency. Cochrane Database Syst Rev. 2011;16:17–43. 10.1002/14651858.CD006768.pub2 PMC4278667 21412896

[13] Scheiman MM , Cooper JS , Mitchell GL , Land P , Cotter SA , Borsting E , et al. A survey of treatment modalities for convergence insufficiency. Optom Vis Sci. 2002;79:151–157. 11913841 10.1097/00006324-200203000-00009

[14] Salus University . CITT Study Procedures Manual [Internet]. Elkins Park (PA): Salus University, Pennsylvania College of Optometry; 2024. https://u.osu.edu/citt/manual‐of‐procedures/

[15] Convergence Insufficiency Treatment Trial (CITT) Study Group . The convergence insufficiency treatment trial: design, methods, and baseline data. Ophthalmic Epidemiol. 2008;15:24–36. 18300086 10.1080/09286580701772037 PMC2782898

[16] Clark TY , Clark RA . Convergence Insufficiency Symptom Survey scores for reading versus other near visual activities in school‐age children. Am J Ophthalmol. 2015;160:905–912. 26275474 10.1016/j.ajo.2015.08.008

[17] Wajuihian SO . Correlations between clinical measures and symptoms: report 2: accommodative and vergence measures with symptoms. Aust J Optom. 2021;14:142–155. 10.1016/j.optom.2020.06.008 PMC8093547 32883648

[18] Pang Y , Tan QQ , Gabriel H , Block SS , Wang J . Application of the convergence insufficiency symptom survey in oculomotor dysfunction and accommodative insufficiency. Optom Vis Sci. 2021;98:976–982. 34393204 10.1097/OPX.0000000000001756

[19] CITT‐ART Investigator Group . Treatment of symptomatic convergence insufficiency in children enrolled in the convergence insufficiency treatment trial‐attention and reading trial: a randomized clinical trial. Optom Vis Sci. 2019;96:825–835. 31651593 10.1097/OPX.0000000000001443 PMC6855327

[20] Talasan H , Scheiman M , Li X , Alvarez TL . Disparity vergence responses before versus after repetitive vergence therapy in binocularly normal controls. J Vis. 2016;16. 10.1167/16.1.7 PMC4743712 26762276

[21] Ip JM , Robaei D , Rochtchina E , Mitchell P . Prevalence of eye disorders in young children with eyestrain complaints. Am J Ophthalmol. 2006;142:495–497. 16935600 10.1016/j.ajo.2006.03.047

[22] Pavel IA , Bogdanici CM , Donica VC , Anton N , Savu B , Chiriac CP , et al. Computer vision syndrome: an ophthalmic pathology of the modern era. Medicina. 2023;59:412. 10.3390/medicina59020412 36837613 PMC9961559

[23] Rosenfield M . Computer vision syndrome: a review of ocular causes and potential treatments. Ophthalmic Physiol Opt. 2011;31:502–515. 21480937 10.1111/j.1475-1313.2011.00834.x

[24] Argiles M , Quevedo‐Junyent L , Graham E . Topical review: optometric considerations in sports versus E‐sports. Percept Mot Skills. 2022;129:731–746. 35084253 10.1177/00315125211073401

[25] Akarsu S , Dane S . Athletes have faster eye‐hand visual reaction times and higher scores on visuospatial intelligence than nonathletes. Turk J Med Sci. 2009;39:871–874.

[26] Omar R , Kuan YM , Zuhairi NA , Manan FA , Knight VF . Visual efficiency among teenaged athletes and non‐athletes. Int J Ophthalmol. 2017;10:1460–1464. 28944208 10.18240/ijo.2017.09.20 PMC5596234

[27] Alvarez TL , Vicci VR , Alkan Y , Kim EH , Gohel S , Barrett AM , et al. Vision therapy in adults with convergence insufficiency: clinical and functional magnetic resonance imaging measures. Optom Vis Sci. 2010;87:E985–E1002. 21057347 10.1097/OPX.0b013e3181fef1aa PMC3134155

[28] Alvarez TL , Scheiman MM , Santos EM , Morales C , Yaramothu C , D'Antonio‐Bertagnolli JV , et al. Clinical and functional imaging changes induced from vision therapy in patients with convergence insufficiency. Proc IEEE Eng Med Biol Soc. 2019;2019:104–109. 10.1109/EMBC.2019.8857163 31945855

[29] Scheiman MM , Talasan H , Mitchell GL , Alvarez TL . Objective assessment of vergence after treatment of concussion‐related CI: a pilot study. Optom Vis Sci. 2017;94:74–88. 27464574 10.1097/OPX.0000000000000936 PMC5182092

[30] Scheiman M , Talasan H , Alvarez TL . Objective assessment of disparity vergence after treatment of symptomatic convergence insufficiency in children. Optom Vis Sci. 2019;96:3–16. 30570596 10.1097/OPX.0000000000001320 PMC6305249

[31] Scheiman MM , Cotter SA , Mitchell GL , Kulp MT , Rouse MW , Hertle R , et al. Randomized clinical trial of treatments for symptomatic convergence insufficiency in children. Arch Ophthalmol. 2008;126:1336–1349. 18852411 10.1001/archopht.126.10.1336 PMC2779032

[32] Scheiman MM , Ciuffreda KJ , Thiagarajan P , Tannen B , Ludlam DP . Objective assessment of vergence and accommodation after vision therapy for convergence insufficiency in a child: a case report. Optom Vis Sci. 2014;2:7–12.

[33] Scheiman M , Mitchell L , Cotter S , Kulp MT , Cooper J , Rouse M , et al. A randomized clinical trial of vision therapy/orthoptics versus pencil pushups for the treatment of convergence insufficiency in young adults. Optom Vis Sci. 2005;82:E583–E595. 10.1097/01.opx.0000171331.36871.2f 16044063

[34] Scheiman M , Chase C , Borsting E , Mitchell GL , Kulp MT , Cotter SA . Effect of treatment of symptomatic convergence insufficiency on reading in children: a pilot study. Clin Exp Optom. 2018;101:585–593. 29577409 10.1111/cxo.12682

[35] Van Leeuwen AF , Westen MJ , Van Der Steen J , De Faber JTHN , Collewijn H . Gaze‐shift dynamics in subjects with and without symptoms of convergence insufficiency: influence of monocular preference and the effect of training. Vision Res. 1999;39:3095–3107. 10664807 10.1016/s0042-6989(99)00066-8

[36] Faul F , Erdfelder E , Lang AG , Buchner A . G*power 3: a flexible statistical power analysis program for the social, behavioral and biomedical sciences. Behav Res Methods. 2007;39:175–191. 17695343 10.3758/bf03193146

[37] Argilés M , Gispets J , Lupón N , Sunyer‐Grau B , Rovira‐Gay C , Pérez‐Ternero M , et al. Impact of strabismus and binocular dysfunctions in the developmental eye movement test and test of visual perception skills: a multicentric and retrospective study. Aust J Optom. 2023;16:277–283. 10.1016/j.optom.2023.04.002 PMC10518761 37142504

[38] Scheiman MM , Cotter SA , Rouse MW , Mitchell GL , Kulp MT , Cooper JS , et al. Randomised clinical trial of the effectiveness of base‐in prism reading glasses versus placebo reading glasses for symptomatic convergence insufficiency in children. Br J Ophthalmol. 2005;89:1318–1323. 16170124 10.1136/bjo.2005.068197 PMC1772876

[39] Rosenfield M , Ciuffeda KJ , Ong E , Super S . Vergence adaptation and the order of clinical vergence range testing. Optom Vis Sci. 1995;72:219–223. 7609946 10.1097/00006324-199504000-00001

[40] Fray KJ . Fusional amplitudes: exploring where fusion falters. Am Orthopt J. 2013;63:41–54. 24141750 10.3368/aoj.63.1.41

[41] Vinuela‐Navarro V , Goset J , Aldaba M , Mestre C , Rovira‐Gay C , Cano N , et al. Eye movements in patients with post‐COVID condition. Biomed Opt Express. 2023;14:3936–3949. 37799689 10.1364/BOE.489037 PMC10549724

[42] Rovira‐Gay C , Mestre C , Argiles M , Vinuela‐Navarro V , Pujol J . Feasibility of measuring fusional vergence amplitudes objectively. PLoS One. 2023;18:e0284552. 10.1371/journal.pone.0284552 37141181 PMC10159156

[43] Santos EM , Yaramothu C , Alvarez TL . Comparison of symmetrical prism adaptation to asymmetrical prism adaptation in those with normal binocular vision. Vision Res. 2018;149:59–65. 29940191 10.1016/j.visres.2018.06.004 PMC6174079

[44] Jenewein EC , Cotter S , Roberts T , Kulp M , Mitchell G , Jones‐Jordan LA , et al. Vergence/accommodative therapy for symptomatic convergence insufficiency in children: time course of improvements in convergence function. Ophthalmic Physiol Opt. 2023;43:105–115. 36271753 10.1111/opo.13062 PMC9798873

[45] Scheiman M , Yaramothu C , Alvarez TL . Changes in the disparity vergence main sequence after treatment of symptomatic convergence insufficiency in children. J Eye Mov Res. 2019;12:4–16. 10.16910/jemr.12.4.6 PMC7079725 32190204

[46] Sangoi A , Scheiman M , Yaramothu C , Santos EM , Gohel S , Alvarez TL . Convergence insufficiency neuro‐mechanism adult population study: phoria adaptation results. Invest Opthalmol Vis Sci. 2021;62:19. 10.1167/iovs.62.10.19 PMC8374988 34406329

[47] Dennis M , Spiegler BJ , Juranek JJ , Bigler ED , Snead OC , Fletcher JM . Age, plasticity and homeostasis in childhood brain disorders. Neurosci Biobehav Rev. 2013;37:2760–2773. 24096190 10.1016/j.neubiorev.2013.09.010 PMC3859812

[48] Jang J , Tai‐Hyung K , Moon H . Effectiveness of vision therapy in school children with symptomatic convergence insufficiency. J Ophthalmic Vis Res. 2017;12:187–192. 28540011 10.4103/jovr.jovr_249_15 PMC5423373

[49] Erkelens IM , Bobier WR . Asymmetries between convergence and divergence reveal tonic vergence is dependent upon phasic vergence function. J Vis. 2017;17. 10.1167/17.5.4 28505662

[50] Erkelens IM , Bobier WR . Reflexive fusional vergence and its plasticity are impaired in convergence insufficiency. Invest Ophthalmol Vis Sci. 2020;61:ARVO E‐Abstract 21. 10.1167/iovs.61.10.21 PMC7441356 32780865

[51] Antona B , Barrio A , Barra F , Gonzalez E , Sanchez I . Repeatability and agreement in the measurement of horizontal fusional vergences. Ophthalmic Physiol Opt. 2008;28:475–491. 18761485 10.1111/j.1475-1313.2008.00583.x

[52] Group CITTS . Long‐term effectiveness of treatments for symptomatic convergence insufficiency in children. Optom Vis Sci. 2009;86:1096–1103. 19668097 10.1097/OPX.0b013e3181b6210f PMC2780441

[53] Lança CC , Rowe FJ . Measurement of fusional vergence: a systematic review. Strabismus. 2019;27:88–113. 30821611 10.1080/09273972.2019.1583675

[54] Pediatric Eye Disease Investigator Group . Home‐based therapy for symptomatic convergence insufficiency in children. Optom Vis Sci. 2016;93:1457–1465. 27575992 10.1097/OPX.0000000000000975 PMC5118058

[55] Wesson MD . Normalization of prism bar vergences. Am J Optom Physiol Opt. 1982;59:628–634. 7137301 10.1097/00006324-198208000-00002

[56] Goss DA , Becker E . Comparison of near fusional vergence ranges with rotary prisms and with prism bars. J Am Optom Assoc. 2011;82:104–107. 10.1016/j.optm.2010.09.011 21144803

