# Supplementary material for: Objective evaluation of fusional vergence after a vision therapy protocol in typical binocular vision
Source: Ophthalmic Physiol Opt. 2025 May 24;45(5):1173–85. doi: 10.1111/opo.13528 (PMC12153028; doi:10.1111/opo.13528)
Supplement: Supplementary file 1 — Data S1. [file OPO-45-1173-s001.docx]

Supplementary

Table S1. Mean (± SD) of baseline data. Δ = prism diopters; NPC = near point of convergence; BAF = binocular accommodation facility; cpm = cycles per minute

| **Optometric Measures (40 cm)** | **Control Group** | | | **Experimental Group** | | | **ANOVA repeated measures**  **(time per group)** |
| --- | --- | --- | --- | --- | --- | --- | --- |
|  | **Mean ± SD**  **Evaluation 1** | **Mean ± SD**  **Evaluation 2** | **Mean ± SD**  **Evaluation 3** | **Mean ± SD**  **Evaluation 1** | **Mean ± SD**  **Evaluation 2** | **Mean ± SD**  **Evaluation 3** |  |
|  |  |  |  |  |  |  |  |
|  |  |  |  |  |  |  |  |
| **Near Phoria (**Δ**)** | 0.06±1.65 | 0.31±3.02 | 2.00±3.11 | -0.93±5.20 | -0.87±4.91 | 2.87±5.05 | F(1.311, 39.319) = 1.582, p = 0.220, ŋ2 = 0.050 |
| **NPC break (cm)** | 9.43±5.04 | 7.62±2.21 | 6.50±1.21 | 6.25±0.68 | 6.25±1.00 | 6.25±0.77 | F(1.272, 38.172) = 4.268, p = 0.037*, ŋ2 = 0.125 |
| **NPC recovery (cm)** | 11.03±5.34 | 9.21±3.10 | 7.75±1.87 | 7.50±1.36 | 7.25±1.00 | 7.43±1.09 | F(1.413, 42.383) = 4.379, p = 0.030*, ŋ2 = 0.127 |
| **BAF**  **(cpm)** | 11.40±2.44 | 11.00±1.95 | 12.62±2.19 | 10.53±3.07 | 11.56±3.32 | 14.06±2.82 | F(1.625, 48.743) = 2.695, p = 0.088, ŋ2 = 0.082 |
| **Vergence facility (cpm)** | 17.43±5.84 | 19.09±6.51 | 19.12±9.56 | 18.34±5.70 | 21.87±4.60 | 16.31±6.87 | F(2, 60) = 3.259, p = 0.058, ŋ2 = 0.098 |

Table S2. Mean (± SD) of baseline data. Δ = prism diopters; NPC = near point of convergence; BAF = binocular accommodation facility; cpm = cycles per minute

| **Optometric Measures (40 cm)** | **Mean ± SD**  **Evaluation 3** | **Mean ± SD**  **Evaluation 4** | **Mean ± SD**  **Evaluation 5** | **ANOVA repeated measures**  **(time)** | **Mean ± SD**  **Evaluation 6** | **T student parellat // Wilcoxon** |
| --- | --- | --- | --- | --- | --- | --- |
| **Near Phoria (**Δ**)** | 2.00±3.11 | 1.06±4.00 | 1.25±3.25 | F(2, 30) = 0.649, p = 0.530, ŋ2 = 0.041 | 0.56±3.46 | t(15) = 0.573, p = 0.575 |
| **NPC break (cm)** | 6.50±1.21 | 6.50±1.21 | 6.06±0.25 | F(1.37, 20.68) = 0.979, p = 0.362, ŋ2 = 0.061 | 0.56±3.46 | Z = -1.00, p = 0.317 |
| **NPC recovery (cm)** | 7.75±1.87 | 7.65±1.49 | 7.06±0.25 | F(1.33, 20.05) = 1.081, p = 0.332, ŋ2 = 0.067 | 7.12±0.34 | Z = -1.00, p = 0.317 |
| **BAF**  **(cpm)** | 12.62±2.19 | 20.12±33.41 | 13.87±3.53 | F(1.02, 15.34) = 0.686, p = 0.424, ŋ2 = 0.044 | 14.84±2.78 | t(15) = -1.155, p = 0.266 |
| **Vergence facility (cpm)** | 19.12±9.56 | 22.06±8.86 | 23.15±8.16 | F(2, 30) = 3.838, p = 0.033, ŋ2 = 0.204 | 25.65±5.60 | t(5) = -1.685, p = 0.113 |
